# Sb and N Incorporation Interplay in GaAsSbN/GaAs Epilayers near Lattice-Matching Condition for 1.0–1.16-eV Photonic Applications

**DOI:** 10.1186/s11671-017-2129-2

**Published:** 2017-05-18

**Authors:** V. Braza, D. F. Reyes, A. Gonzalo, A. D. Utrilla, T. Ben, J. M. Ulloa, D. González

**Affiliations:** 1 Departamento de Ciencia de los Materiales e IM y QI, Universidad de Cádiz, 11510 Puerto Real, Cádiz, Spain; 2 Institute for Systems based on Optoelectronics and Microtechnology (ISOM), Universidad Politecnica de Madrid, Ciudad Universitaria s/n, 28040 Madrid, Spain

**Keywords:** GaAsSbN, Dilute nitride semiconductor, Structural and optical characterization, 81.05.Ea (III-V semiconductors), 81.15.Hi (molecular beam epitaxy), 68.55.Nq composition and phase identification, 71.20.Nr Semiconductor compounds

## Abstract

As promising candidates for solar cell and photodetection applications in the range 1.0–1.16 eV, the growth of dilute nitride GaAsSbN alloys lattice matched to GaAs is studied. With this aim, we have taken advantage of the temperature gradient in the molecular beam epitaxy reactor to analyse the impact of temperature on the incorporation of Sb and N species according to the wafer radial composition gradients. The results from the combination of X-ray diffraction (XRD) and energy-dispersive X-ray spectroscopies (EDS) show an opposite rate of incorporation between N and Sb as we move away from the centre of the wafer. A competitive behaviour between Sb and N in order to occupy the group-V position is observed that depends on the growth rate and the substrate temperature. The optical properties obtained by photoluminescence are discussed in the frame of the double-band anticrossing model. The growth conditions define two sets of different parameters for the energy level and the coupling interaction potential of N, which must be taken into account in the search for the optimum compositions 1–1.15-eV photonic applications.

## Background

Dilute III-N-V semiconductor alloys have attracted a great deal of attention due to its possible use in GaAs-based photonic devices such as solar cells and photodetectors [1, 2]. Their abilities, like being grown lattice-matched to GaAs and the possibility of tuning their bandgap in the range 0.9 to 1.5 eV, are potentially beneficial to these devices. In the photovoltaic field, its incorporation in multi-junction solar cells (MJSCs) based on III–V semiconductor materials is paying huge attention, [3–5] since they are the most efficient sort of solar cells [6]. In fact, it has been shown that using a 1-eV layer in conventional triple junction SC, replacing the Ge or enabling four junction SC architectures, would allow improving notably their efficiency [7, 8]. In photodetectors, one of the key challenges is the achievement of high-efficiency near-infrared photodetectors. Nowadays, the switchover in space communications from radiofrequency to optical in the near-infrared region (0.9–1.6 μm) is becoming a reality, being 1064 nm (1.16 eV) the most widely used wavelength. Although some optical devices have already been used by different space agencies [9, 10], further improvement is still needed.

Amongst the possible III–V-based dilute nitride materials, GaAsSbN system is a promising one for such technologies [11, 12]. This system can be grown with narrow band gap staying lattice matched to GaAs [13] while it also adjusts independently both conduction and valence-band energies by controlling the N and Sb content, respectively [14, 15]. Besides, it presents better thermal stability by having only one element of group III, and it needs a lower N composition to reach for the same band gap (and therefore it shows fewer N-related defects) than the most intensely studied GaInAsN material [16]. Other advantages are the absence of In-related defects and a more efficient incorporation of N atoms [17, 18]. For certain, GaAsSbN has already been demonstrated as a promising material for near-infrared photodetectors [19–22] and solar cells [23–26].

However, despite the intense scientific effort, this technology is still suffering from the difficulty for growing high-quality layers. The presence of three group-V elements adds a complexity in the GaAsSbN growth causing technical hitches in the compositional control of these layers. Certainly, the effect of the concomitant incorporation of Sb and N or the impact of the growth parameters in their incorporation is still not well understood [12, 27, 28]. This research focuses on the growth control of high-quality GaAsSbN layers grown at 2 ML/s for photonic devices in the bandgap range of 1–1.16 eV. The use of growth rates higher than the one commonly applied in GaAs-based compounds (~1 ML/s) has been proved to have a positive impact in other N-containing structures, probably by reducing the composition modulation as a result of a lower diffusion of N and Sb atoms on the growth surface [29]. Moreover, we have taken advantage of the radial composition drift of the MBE growth, induced by the substrate temperature gradient, to multiply the range of the compositions of Sb and N obtained in each growth. The simultaneous incorporation of Sb and N was analysed using high-resolution X-ray diffraction (HR-XRD), energy-dispersive X ray spectroscopy (EDX) and different techniques of transmission electron microscopy. The results show the existence of a competition between Sb and N species, in disagreement with previous studies reporting an enhanced incorporation of both elements under their simultaneous presence [17, 30–32]. The analysis by photoluminescence (PL) was realized in the frame of the double-band anticrossing (DBAC) model, and the parameters referred to N are compared with the bibliography.

## Methods

### Materials

The GaAs_1 − *x* − *y*_Sb_*x*_N_*y*_ epilayers were grown by solid source molecular beam epitaxy (MBE) on Si-doped (100) GaAs substrates. The samples of 10 × 10 mm are composed by 250-nm-thick n-doped GaAs buffer layer and a thick active layer of GaAsSbN (200–750 nm) covered with 50 nm of GaAs. A standard effusion cell was used as the Sb_4_ source, while active N was generated from a radio frequency (RF) plasma source with a 0.1 sccm flow of pure N_2_ (6N). Quaternary samples C1, C2 and C3 were grown at 470 °C with a fixed N flux and different Sb fluxes (see Table 1). A high growth rate of 2 ML/s is chosen in these N- and Sb-containing structures since it has been shown to strongly improve the PL emission in other samples containing the quaternary alloy [29]. A GaAsN sample (C0) was grown as a reference. In addition, another sample (C3L) was grown at the standard growth rate of 1 ML/s and with half the N flux and the same Sb flux than C3 for comparison. In addition, all the samples were later annealed at 850 °C during 30 s and compared to the as-grown counterparts. The growth rates (*V*_g_), the optical emission detection voltage (OED), directly proportional to the amount of active N in the plasma, and the Sb_4_ beam equivalent pressure (BEP) of the samples are indicated in Table 1. The OED values between 3000 and 3050 mV can be considered the same, since the difference is within the resolution allowed by the stabilization conditions of the plasma.

**Table 1:** Nominal growth rate (Vg), N OED and Sb flux

Sample	*V*_g_(ML/s)	N OED (mV)	Sb_4_ BEP (10^−7^ Torr)
C0	1	1260	0
C1	2	3000	2.6
C2	2	3030	1.6
C3	2	3050	1.3
C3L	1	1530	1.3

### Equipment and Techniques

From each sample, different small pieces were taken at different distances from the centre of the wafer for the analysis. The small size of the studied pieces minimizes the effect of the gradients within each piece. First, the samples were characterized by HR-XRD and PL. The lattice parameters of the samples were measured using X-ray diffraction in a Panalytical X’Pert Pro (PANalytical V.B., Almelo, Netherlands) diffractometer equipped with a Ge (220) hybrid monochromator. The photonic emission was evaluated by PL at 15 K using a closed-cycle Helium cryostat, a He–Ne laser as excitation source and a liquid-nitrogen-cooled Ge-detector.

Cross-sectional samples for transmission electron microscopy (TEM) studies were prepared using mechanical thinning followed by ion polishing. Then, crystal quality of the GaAsSbN layers were analysed by diffraction contrast (DC) TEM and annular dark field (ADF) in scanning TEM (STEM) mode with a JEOL-2100 LaB6 (JEOL Ltd, Akishimashi, Tokyo, Japan) microscope operating at 200 kV. The composition of Sb in the GaAsSbN was quantified by EDX spectroscopy with an Oxford Inca Energy-200 detector (Oxford Instruments, Abingdon, UK). Finally, the band gap energies and the N-related parameters were estimated by using the DBAC model.

## Results and Discussion

### Structural and Compositional Characterization

**Fig. 1:**
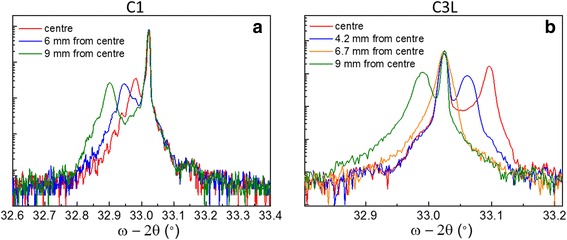
XRD omega-2theta scans around (0 0 4) GaAs reflection of **a** sample C1 and **b** sample C3L at different distances from centre of wafer

In order to obtain a great number of different compositions of GaAs_1 − *x* − *y*_Sb_*x*_N_*y*_ alloys, we take advantage of the temperature gradient present during the MBE growth from the centre of the wafer to its edge. [33, 34]. Previous measurements in our system have given a temperature gradient, which could be considered linear in a first approach, of about −1.25 °C/mm from the centre of the wafer, although it actually becomes stronger as we get closer to the edge. Indeed, the growth temperature is likely the most critical parameter to achieve high-quality GaAsSbN layers, since a balance is required to obtain an efficient and simultaneous Sb and N incorporations [12, 35, 36]. This dependence of the Sb and N incorporation with the substrate temperatures may lead to compositional non-uniformities due to temperature gradients along the wafer diameter during the growth. Thus, we compared the reticular mismatch to GaAs and composition of different samples at different distances from the centre of wafer. Figure 1 shows XRD ω-2*θ* scans around (0 0 4) GaAs reflection at different distances from the centre in C1 and C3L samples (a similar tendency is observed in the rest of the samples). The XRD spectra present a main peak due to GaAs substrate and one secondary peak if the epilayer is not perfectly lattice-matched to the substrate. In general, our GaAs_1 − *x* − *y*_Sb_*x*_N_*y*_ samples did not fulfil the lattice-matching condition and presented one peak close to the GaAs peak. The secondary peak are always moved to the left when we go away from wafer centre, indicating a displacement to lesser tensile or more compressive states. Even so, all samples presented small lattice mismatches, lower than 10^-3^, maintaining the Sb/N ratio close to the GaAs lattice-matching condition, namely, (*x*/*y* ≈ 2.6).

**Fig. 2:**
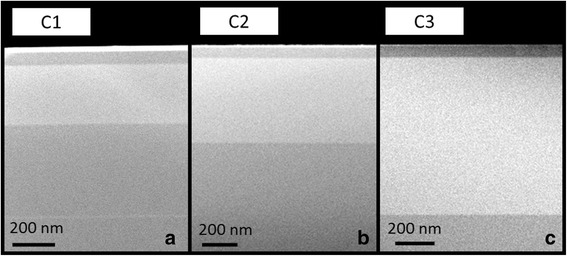
ADF images of samples **a** C1 with 200 nm of active layer, **b** C2 with 400 nm of active layer and **c** C3 with 750 nm of active layer acquired along the [110] zone axis

ADF STEM analyses were developed instead of high-angle ADF since the micrograph intensity contrast was very low in our samples, as result of the low Sb and N contents. In ADF images, the GaAsSbN layers appear brighter than the GaAs, mainly due to the N presence [37]. No clear contrasts due to, for example, composition modulation or vertical segregation phenomena were exhibited in samples along the growth direction as we can see in Fig. 2. Compositional sensitive g200 dark field (DF) and strain sensitive 220 bright field (BF) imaging in DCTEM conditions are also used to analyse the structural quality of the GaAsSbN layers. DCTEM analyses revealed that samples are homogenous and free from dislocations or any other type of extended defects so that all samples experimented pseudomorphic growth. This fact is indicative of a Sb/N ratio close to the lattice-matching condition to GaAs, as indicated previously, taking into account the large thickness of some of the samples (up to 750 nm).

**Fig. 3:**
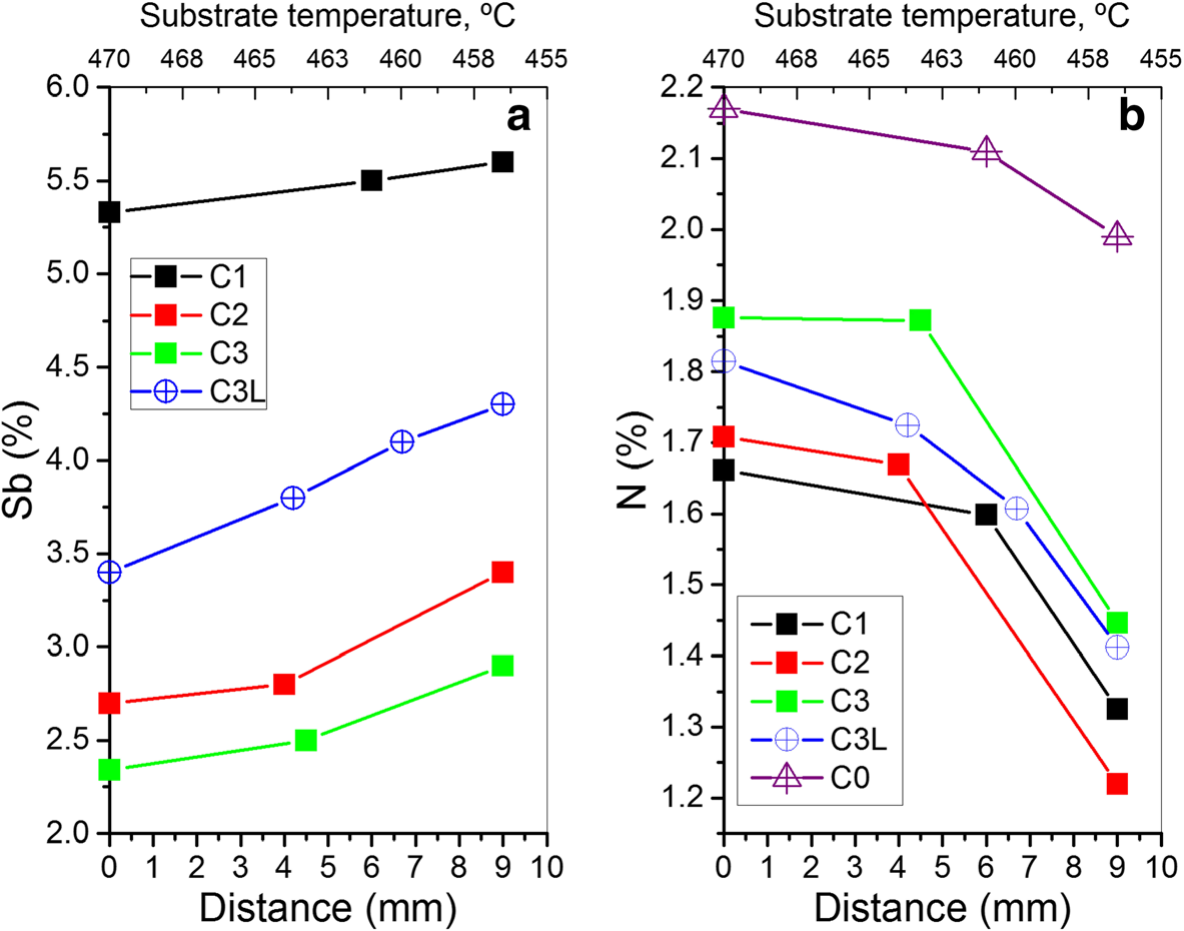
Plots of the average composition of **a** Sb and **b** N versus distance from centre of the wafer of all samples. Top scale is the estimated growth substrate temperature

The average Sb content was statistically computed from pointed EDX spectra taken out at different regions of each TEM sample. These EDX measurements of the Sb content follow a normal distribution that confirmed the nonexistence of any compositional gradient. The averaged measurements of Sb contents are displayed versus distance from the wafer centre in Fig. 3a and revealed that the Sb incorporation increases toward the edge of wafer according to a decrement of substrate temperature. The inverse relation between the growth temperature and the incorporation of Sb into GaAsSb was first demonstrated by Chang et al. [38] and is generally agreed on in the literature. This behaviour may be explained by a preferential incorporation of Sb over As at low temperatures as a result of the lower atomization energy (203.6 kcal/mole for Sb_4_ versus 252 kcal/mole for As_4_) and higher sublimation energy (49.4 kcal/mole for Sb_4_ versus 36.6 kcal/mole for As_4_) of Sb_4_ compared to As_4_ that facilitates a longer lifetime presence of Sb on the surface [35, 36]. These differences in energies will increase the relative Sb/As incorporation following decrease in the growth temperature. However, at higher temperatures, the more efficient dissociation of As_4_ that allow it to become more competitive with the Sb, as well as the increased rate of Sb evaporation, cause a significant reduction in the Sb/As incorporation ratio.

The determination of the average N content is not possible by EDX due to its low atomic number. To overcome this handicap and calculate the average N contents, we have combined the results of lattice parameters obtained from the XRD data (using the zero plastic relaxation condition as confirmed by TEM) and the Sb measurements by means of EDX. A similar procedure has been applied for the case of GaInNAsSb layers grown by MBE [39]. The N content versus the distance from the wafer centre is depicted in the marked line chart of Fig. 3b. From the results, we can first stand out a relationship between the N and Sb incorporation. The N incorporation in the presence of Sb is reduced compared to the case of GaAsN. If we compare the values of the wafer centre (i.e. distance zero in the graph) of samples C0 and C3L (both grown at 1 ML/s), we see a strong reduction in the N content in sample C3L despite having larger OED, due to the fact that a 3.4% Sb has been incorporated. Moreover, if we focus on data of the wafer centre of the quaternary samples grown at the same growth rate, the increase of the Sb contents leads to a reduction in the N incorporation. The samples with higher Sb composition exhibit lesser N content. This could appear in contradiction with the widespread belief of a general enhancement of the N incorporation with the presence of Sb [17, 30] and vice versa [18]. However, it was reported in the literature that the concomitant presence of Sb and N could manifest an ambivalent behaviour depending on the growth conditions [27, 40, 41]. At low Sb fluxes (<1 10^−8^ Torr) and standard growth rates (1 ML/s), Sb atoms could act like an impurity, strongly competing against N atoms for the As sites and thus reducing its incorporation [42]. At higher fluxes, Sb acts as a surfactant, promoting the N incorporation as Sb is increased [40, 41]. When the Sb fluxes are around 10^−8^ Torr, a transition area between the two behaviors exists and the N seems not to be affected. In our case, with fluxes higher than 10^−7^ Torr, results denote that the faster growth rate delays the transition from impurity to surfactant behaviour and, as a consequence, N and Sb atoms remain competing to incorporate in the layer at this flux level. The change of the Sb surfactant behaviour according to the growth rate has been recently reported by the authors. Indeed, GaAsSb(N) capping layers (CLs) could play antagonistic roles regarding the InAs QD decomposition depending on their growth rate. While at common growth rates (1 ML/s), GaAsSb(N) CLs prevent QD dissolution; at high growth rates of 2 ML/s, the Sb does not protect the QDs but even intensifies their erosion [43]. Moreover, the growth rate also affects the Sb incorporation since sample C3 grown at 2 ML/s presents significantly more than half the Sb content of sample C3L, grown at 1 ML/s with the same Sb flux. The N flux was reduced to half along with the growth rate, and the incorporated N content is virtually the same in both samples, indicating that N incorporation is inversely proportional to the growth rate, as has been reported for GaAsN and InGaAsN [44, 45]. Nevertheless, the situation is different for the Sb: the incorporated Sb is not doubled when reducing the growth rate to half and keeping the same Sb flux. This indicates a more efficient Sb incorporation at 2 ML/s than at 1 ML/s. This growth rate features, as far as we know, have not been reported previously. From results, we can secondly stand out an N incorporation dependence on the temperature.

By focusing on the behaviour of N incorporation with respect to distance in Fig. 3b, it can be denoted that the N content undergoes a decrease as we move toward the wafer edge. Note that the N content remains almost constant only declining beyond 7 mm away from the centre. This decrease is of greater magnitude than what it could be related to changes in the growth temperature. Results for sample C0, corresponding to a GaAsN single layer using similar conditions, suggest that the incorporation of N has a weak reliance on growth temperature since the N content decay at the external region of the wafer is very low. Therefore, the reduced N content in the edge of the wafer in the GaAsSbN samples is largely due to the higher Sb content in these places. Certainly, the N incorporation in GaAsSbN layers is more complex than in GaAsN ones, where the sticking coefficient of N is almost temperature independent within the range 420–550 °C [46]. Ma et al. reported for GaAsSbN layers an enhanced N incorporation occurs with the presence of Sb under molecular-N_2_-rich conditions, and the opposite under atomic-N-rich conditions, where Sb and atomic-N compete for the same positions [35, 47]. Moreover, the incorporation of N is greatly amplified by increasing temperature under atomic-N-rich conditions and becomes nearly temperature independent in the case of molecular-N_2_-rich conditions. Our results indicate that, in our growth conditions, atomic-N becomes the dominant reactive species that competes with Sb for anionic sites. As we move away from the centre, the growth temperature is lower and the presence of Sb at surface increases. Both effects, the lower growth temperature together with the higher coverage by Sb atoms, add up leading to a diminution of the sticking coefficient of N [12] that would explain the N composition gradient at the edge of the wafer.

### Optical Characterization

**Fig. 4:**
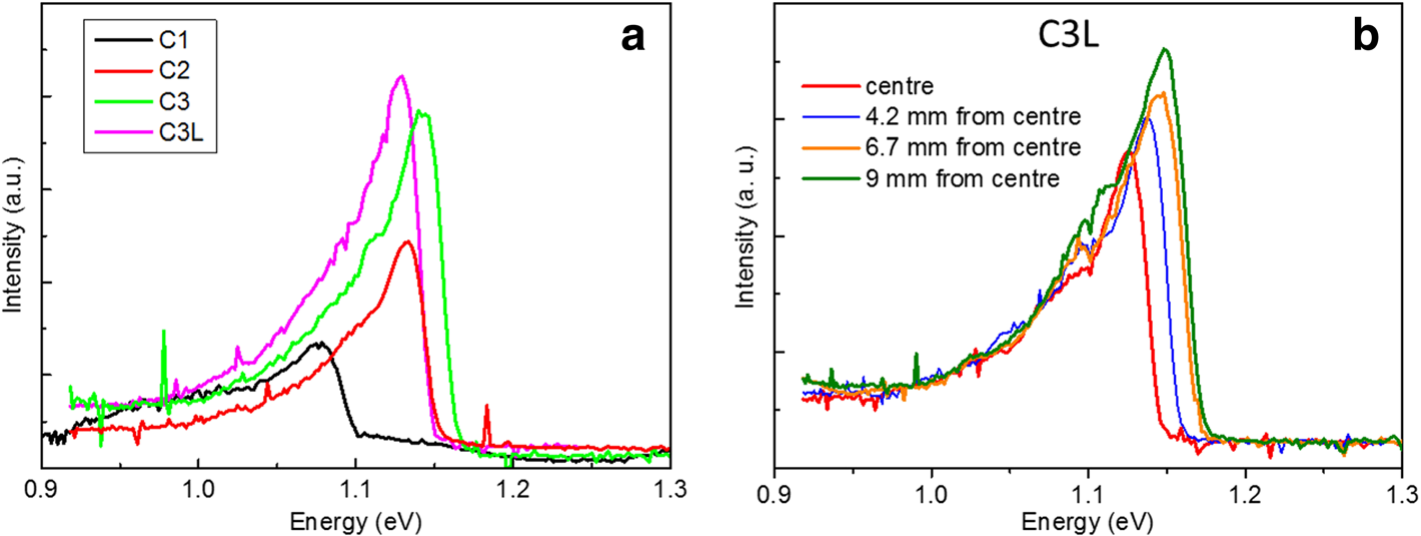
15K PL spectra of **a** all samples at the center of wafers and **b** sample C3L at different distances from centre of wafer

Figure 4a contains the low-temperature PL spectra of all samples of the wafer centre. The graph shows an intensity rising when increasing the thickness of the samples due to a greater volume of active layer. In the same way, a series of PL spectra were taken for each sample at different distances from the centre of the wafer in the very same pieces analysed before. Figure 4b shows the case for the sample C3L. As we can see, a blueshift occurs as we move away from the centre while the peak intensity is increasing, fact that happens in all the samples. This indicates that not only the emission wavelength but also the integrated intensity is mainly determined by the N content: the lower the N content, the more efficient the PL emission. Remarkably, no correlation between the residual strain and the PL efficiency is observed, even in the thicker samples of 750 nm. This means that in the present range of lattice mismatch (below 10^−3^), the N content and not the residual strain is the main factor determining the optical quality of the structures.

In order to understand the PL behaviour in GaAsSbN alloys, the DBAC model [48, 49] is generally used. Briefly, DBAC model is a combination between valence-band anticrossing (VBAC) and conduction band anticrossing (CBAC) models [50]. The CBAC model was developed to explain the pressure and composition dependencies of the bandgap in III–V diluted nitride alloys. In this model, when the impurity species have a much greater electronegativity than that of the host anion, the defect states of impurity atoms as N are often located near the conduction band edge of the host. The result is an anticrossing interaction between highly localized states of substitutional highly electronegative atoms and the extended states of the host semiconductor matrix [51]. For a generic nitride AB_1 − *y*_N_*y*_ alloy, this interaction forms two subbands, named *E*_*c*_^+^ and *E*_*c*_^−^, given by:

1 $$ {E}_C^{\mp }=\frac{1}{2}\left\{{E}_N+{E}_{C B}\mp \sqrt{{\left({E}_{C B}-{E}_N\right)}^2+4{C}_N^2 y}\right\} $$

where *E*_CB_ is the lowest conduction band of the host material and *E*_N_ is the energy of the localized states derived from the N atoms related to CB of the GaAs. The coupling between the localized states and the band states of the host is described by the hybridization parameter *C*_N_.

On the other hand, the defect states of large-sized impurities as Bi or Sb with first ionization energies less than that of the host anion stand near the valence-band edge of the host semiconductor and a hybridization between the two results in a modification of the valence band [50]. For a GaAs_1 − *x*_Sb_*x*_ alloy, this interaction forms two subbands, named *E* ^+^ and *E* ^−^, given by:

2 $$ {E}_V^{\mp }=\frac{1}{2}\left\{{E}_{Sb}+{E}_{V B}\mp \sqrt{{\left({E}_{V B}-{E}_{Sb}\right)}^2+4{C}_{Sb}^2 x}\right\} $$

where *E*_VB_ is the valence band maximum of the GaAsSb and *E*_Sb_ is the energy of the localized Sb level relative to the VB maximum of GaAs. The coupling between the localized states and the band states of the host is described by the hybridization parameter *C*_Sb_. The virtual crystal approximation [52] is used to describe the change of valence and conduction band for GaAsSbN layers. Thus, the energies *E*_VB_ and *E*_CB_ are calculated by equation 3 as [48]:

3 $$ {E}_{CB, VB}={E}_{GaAs}+\varDelta {E}_{CB, VB}\cdot x $$

being *E*_GaAs_ the CB or VB of GaAs and Δ*E*_*CB,VB*_ is the CB or VB offset between GaAs and GaSb.

In the GaAsSbN alloy, both Sb and N produce anticrossing interactions that affect the valence band and conduction band, respectively. These interactions are independent, and they do not affect each other, so CB and VB can be independently manipulated by controlling the N and Sb concentrations [48]. The model combines the conduction BAC and valence BAC models where the bandgap *E*_0_ is

4 $$ {E}_0={E}_C^{-}-{E}_V^{+} $$

**Fig. 5:**
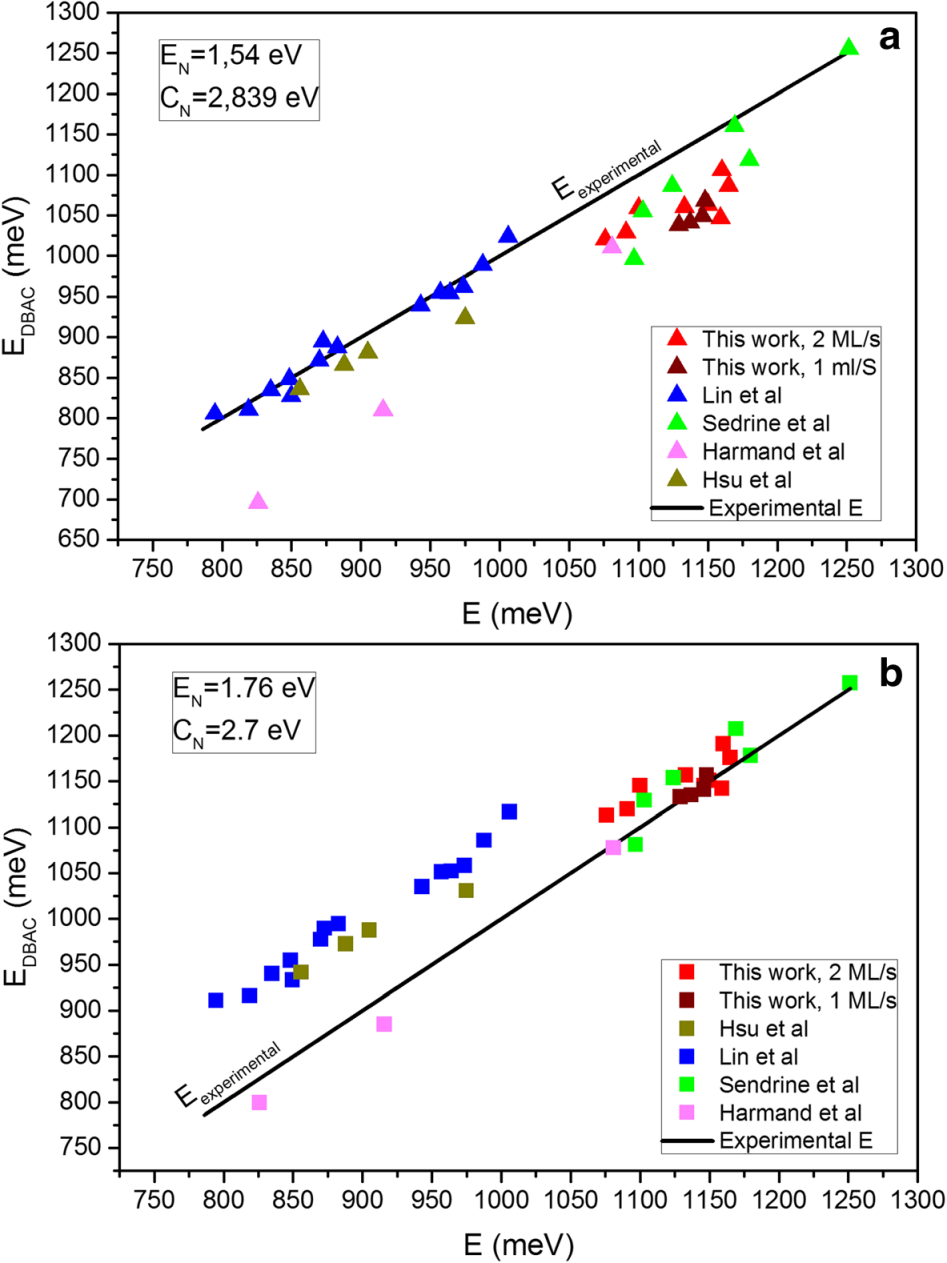
Plots of the calculated DBAC energies versus the experimental ones by using **a** Lin’s and **b** Sedrine’s parameters for *E*_N_ and *C*_N_ for different as-grown samples. Two identifiable groups are clearly visualized

We have assumed that the energy parameters of the GaAsSb are validated so *E*_*CB*,GaAs_ = 1.42 eV, *ΔE*_*CB*_ = 0.2 eV, *E*_*VB,*GaAS_ = 0 eV, *ΔE*_*VB*_ = 0.5 eV. *E*_Sb_ = −1 eV and *C*_Sb_ = 1.05 eV [49, 50, 53]. However, the values of *E*_N_ and *C*_N_ are still disputable matters. The *E*_N_ and *C*_N_ values of 1.65 and 2.7 eV, obtained for the GaAsN [54] case, have been suggested [48]. Other pairs of values for as-grown samples have been proposed as the best fit parameters for *E*_N_ and *C*_N_ such as Lin (1.540 and 2.839 eV) [49], Sedrine (1.76 and 2.7 eV; 1.6 and 2.7 eV) [53, 55] or Zhao (1.65 and 2.68 eV) [56]. The differences among the values, although apparently small, yield big differences in the outcomes. In addition, there are different sets of parameters for the annealed samples: Lin (1.65 and 2.7 eV) [48], Nunna (1.65 and 3.9 eV) [57] or Sedrine (1.8 and 2.7 eV) [55]. In Fig. 5a, the calculated transition energies using the DBAC model of GaAsSbN alloys from this work and other authors are plotted with the parameters proposed by Lin et al. [49], *E*_N_ = 1.540 eV and *C*_N_ = 2.839 eV, versus the experimental energies. The solid line of slope 1 represents the perfect matching between calculated and measured energies. The vertical distance of each point to the line can be associated to the error of the theoretical calculation compared to the measured results. Two different data groups can be clearly appreciated. With these parameters, the calculated energies from Lin et al. [49] and Hsu et al. [58] are in good agreement with their experimental results, but this is not the case for our samples and those of other authors. The best fit to our experimental data is obtained assuming the energy *E*_N_ = 1.76 eV and the hybridization parameter *C*_N_ = 2.7 eV, see Fig. 5b, the same parameters obtained by Sedrine et al. [59] It seems that the DBAC model requires of two sets of parameters in order to account for the experimental band gap energies of GaAsSbN layers.

The differences between both sets of parameters could be explained by differences in the growth conditions that influence on the density of N-related defects such as interstitials, substitutional N pairing or clustering in the samples. Lin et al. already assumed this reason for the high deviation of their as-grown samples with respect to their annealed ones [48]. Indeed, the reduction of N-related defects has been suggested by different authors to explain the blueshift observed in annealed samples [60, 61]. Our fitting parameters are closer to the annealing parameters of Lin et al. indicating that our samples could have a better incorporation of substitutional N atoms and a lower density of N-related defects. Certainly, it is well known that the dilute nitrides grown at temperatures below 480 °C have a better solubility suppressing the concentration fluctuations observed at higher temperatures where the 3D growth condition dominates [46, 61]. At higher temperatures, 3D growth occurs in GaAsN related alloys, characterized by a strong interaction between N atoms that leads to either the formation of N-rich clusters or N segregation [46]. Although the use of surfactants (Sb) could increase those temperatures, the trend must be the same. The samples of Lin and Hsu were grown at 490 °C, and the rest (including our samples) were grown at temperatures below 470 °C. In addition, our samples were grown at faster growth rates that also limit the interaction between N atoms. The growth temperature, the growth rate and even the performance of Sb as surfactant ought to influence in the proportion of N-induced defects in the GaAsSbN layers.

**Fig. 6:**
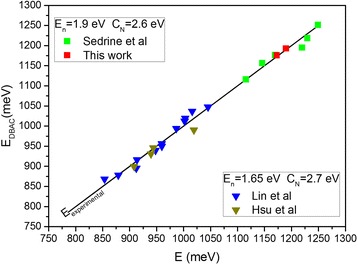
Plot of the calculated DBAC energies versus the experimental ones for different annealed samples by using different sets of parameters for *E*_N_ and *C*_N_

In the case of the annealed samples, our results and others from the literature are plotted in Fig. 6. The graph shows the calculated band gap energies using Lin parameters (*E*_N_ = 1.65 eV, *C*_N_ = 2.7 eV) for the samples grown at higher temperatures (Lin et al. and Hsu et al.) and calculated band gap energies with our fitting parameters (*E*_N_ = 1.9 eV, *C*_N_ = 2.6 eV) for samples grown at lower growth temperatures (our samples and Sedrine et al.). All the annealed samples show a blueshift due to an enhancement in the N distribution and a reduction of the density of punctual defects. In the case of our samples, the blueshifts are in the range of 35–45 meV, while the Lin samples showed higher blueshifts doubling our values. This pointed to a more homogenous structural arrangement in our as-grown samples, likely due to the higher growth rate. This is in agreement with the assumption that atomic-N species are the dominant ones in our growth conditions. In the case where molecular N_2_ species are the main responsible of the N incorporation, the density of N defects, such as N-N split interstitial and (2N_As_)_nn_ clusters, should be higher [37] and the effect of the annealing treatment can lead to higher blueshifts. This confirms the influence of the growth conditions in the parameters of the DBAC model related to the presence of N.

**Fig. 7:**
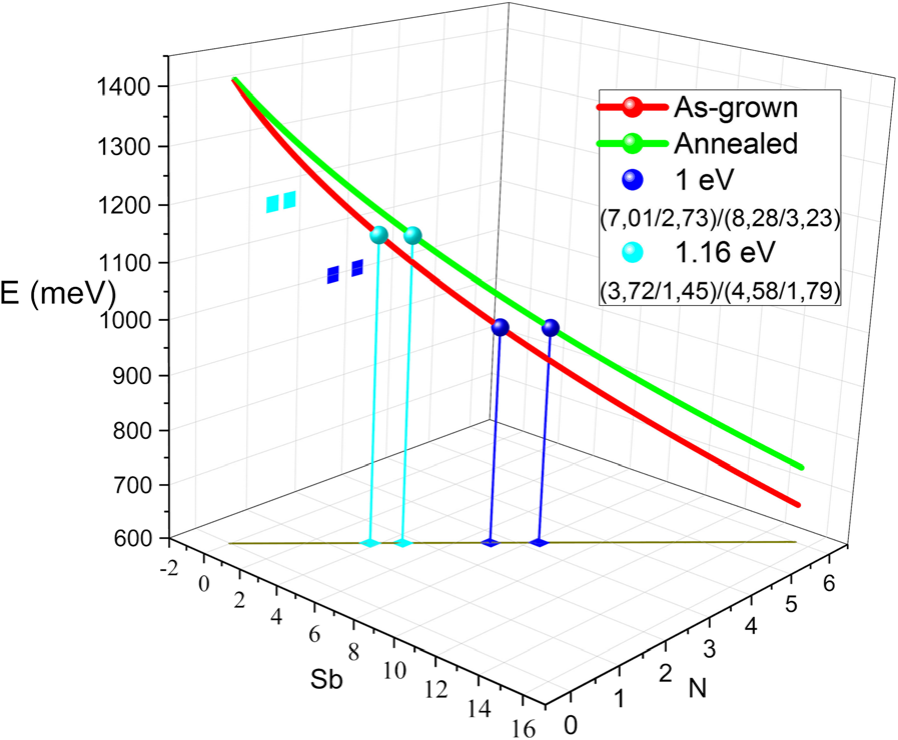
DBAC estimated bandgap for as-grown and annealed GaAsSbN samples with a GaAs lattice-matched condition versus Sb and N contents. The Sb and N compositions for 1.0 and 1.16 eV are estimated

Our fittings manifest a strong influence of the growth conditions in the values of the DBAC parameters of N, *E*_N_ and *C*_N_, due to the different distribution of N species. In order to estimate the suitable compositions for realizing 1 eV solar cells or 1064 nm (1.16 eV) detectors, this fact must be taken into account. Figure 7 displays the estimated energy by DBAC model versus Sb and N contents where the red line is the energy for as-grown samples and the green line the energy for annealed samples in conditions of lattice matching (olive line in the Sb-N plane). Finally, the dots show the contents required for band gap energies of 1 eV (blue dots) and 1.16 eV (cyan dots) for as-grown and annealed samples, respectively, in lattice-matching condition. For 1 eV, the suitable compositions are 7.01% of Sb and 2.73% of N for the as-grown samples and 8.28% of Sb and 3.23% of N for the annealed samples. For 1.16 eV, the suitable composition is 3.72% of Sb and 1.45% of N for the as-grown samples and 4.58% of Sb and 1.79% of N for the annealed samples.

## Conclusions

In summary, GaAsSbN alloys are promising materials for fabricating lattice-matched subcells at 1 eV in MJSCs, as well as for efficient 1064-nm photodetectors. However, the growth conditions have to be precisely controlled since they have a strong effect in the incorporation and spatial distribution of N and Sb and hence in their optical properties. We have studied the influence of temperature gradient in the incorporation of Sb and N, where an antagonist behaviour is observed as we moved away from the centre of the wafer. We have observed a competitive behaviour to occupy the group-V positions between the N and Sb, where the higher Sb content is incorporated, the lower N content is present. The *E*_N_ level and the interaction potential *C*_N_ using the DBAC model are determined for as-grown and annealed samples as *E*_N_ = 1.76 eV and *C*_N_ = 2.7 eV and *E*_N_ = 1.9 eV and *C*_N_ = 2.6 eV, respectively. The comparison with other results from the literature pointed to the existence of two different groups in terms of the BAC parameters required to explain the optical properties of GaAsSbN layers. The discrepancy is attributed to differences in the density of N-related defects due to the different growth conditions.

## Abbreviations

ADF: Annular dark field
BEP: Beam equivalent pressure
BF: Bright field
CBAC: Conduction-band anticrossing model
CLs: Capping layers
DBAC: Double-band anticrossing model
DC: Diffraction contrast
DF: Dark field
EDS: Energy-dispersive X-ray spectroscopies
HR-XRD: High-resolution X-ray diffraction
MBE: Molecular beam epitaxy
MJSCs: Multi-junction solar cells
OED: Optical emission detection voltage
PL: Photoluminescence
QD: Quantum dot
RF: Radio frequency
STEM: Scanning transmission electron microscopy
TEM: Transmission electron microscopy
VBAC: Valence-band anticrossing model
XRD: X-ray diffraction
